# Validity and reliability of the simplified Chinese patient-reported outcomes version of the common terminology criteria for adverse events

**DOI:** 10.1186/s12885-021-08610-0

**Published:** 2021-07-27

**Authors:** Shan-Shan Yang, Lei Chen, Ying Liu, Hai-Jun Lu, Bo-Jie Huang, Ai-Hua Lin, Ying Sun, Jun Ma, Fang-Yun Xie, Yan-Ping Mao

**Affiliations:** 1 Department of Radiation Oncology, Sun Yat-sen University Cancer Center, State Key Laboratory of Oncology in South China, Collaborative Innovation Center for Cancer Medicine, Guangdong Key Laboratory of Nasopharyngeal Carcinoma Diagnosis and Therapy, 651 Dongfeng Road East, Guangzhou, 510060 People’s Republic of China; 2 Nursing Department, Jinan Seventh People’s Hospital, Jinan, China; 3 Department of Radiation Oncology, the Affiliated Hospital of Qingdao University, Qingdao, China; 4 Department of Oncology, the First People’s Hospital of Tianmen in Hubei Province, Tianmen, China; 5 Department of Medical Statistics and Epidemiology, School of Public Health, Sun Yat-sen University, Guangzhou, China

**Keywords:** Patient-reported outcomes, PRO-CTCAE, Chinese, Validity, Reliability

## Abstract

**Background:**

The psychometric properties of the simplified Chinese version of the Patient-Reported Outcomes version of the Common Terminology Criteria for Adverse Events (PRO-CTCAE) have not been assessed. Therefore, we aimed to assess its validity, reliability, and responsiveness.

**Patients and methods:**

A Chinese version of the PRO-CTCAE and the European Organization for Research and Treatment of Cancer Core Quality of Life Questionnaire (QLQ-C30) were distributed to 1580 patients from four cancer hospitals in China. Validity assessments included construct validity, measured by Pearson’s correlations and confirmatory factor analysis (CFA), and known-groups validity, measured by t-tests. The assessment of reliability included internal consistency, measured by Cronbach’s ɑ, and test-retest reliability, measured by the intraclass correlation (ICC). Responsiveness was assessed by standardized response means (SRMs).

**Results:**

Data from 1555 patients who completed the instruments were analyzed. The correlations were high between PRO-CTCAE items and parallel QLQ-C30 symptom scales (*r* > 0.60, *p* < 0.001), except for fatigue (severity: *r* = 0.49). Moreover, CFA showed the PRO-CTCAE structure was a good fit with the data (Root Mean Square Error of Approximation = 0.046). Known-groups validity was also confirmed. Cronbach’s ɑ of all item clusters were greater than 0.9 and the median test-retest reliability coefficients of the 38 items were 0.85 (range = 0.71–0.91). In addition, the SRMs of PRO-CTCAE items were greater than 0.8, indicating strong responsiveness.

**Conclusion:**

The simplified Chinese version of the PRO-CTCAE showed good reliability, validity, and responsiveness.

**Supplementary Information:**

The online version contains supplementary material available at 10.1186/s12885-021-08610-0.

## Introduction

Reporting adverse events (AEs) is mandatory in clinical cancer trials to ensure the safety of patients and identify toxic characteristics of treatments. The National Cancer Institute’s (NCI’s) Common Terminology Criteria for Adverse Events (NCI-CTCAE), which is commonly used in clinical settings, uses terminology consistent with the Medical Dictionary for Medical Affairs [1]. The latest version (version 5.0) of the NCI-CTCAE, released in 2017, contains 837 items that consist of laboratory tests, symptoms, and clinical events, all of which use a 5-point scale that ranges from Grade 1 (mild) to Grade 5 (extremely severe) [2]. The grade of an AE is usually reported by clinicians based on the NCI-CTCAE. However, there is substantial evidence that clinicians are inclined to underestimate the severity of AEs and might miss some of patients’ symptomatic AEs, compared to AEs reported by patients themselves [3–6]. It is very important to obtain information about AEs from the patient’s perspective to supplement symptomatic AEs reported by clinicians. Thus, the NCI developed a patient-centered reporting system to be a companion to the CTCAE, which is named the Patient-Reported Outcomes version of the CTCAE (PRO-CTCAE) [7, 8]. In light of the variability of AEs within and across trials, the PRO-CTCAE is a systematic measurement tool that can effectively capture the patient’s voice in reporting symptomatic AEs in cancer clinical trials. The PRO-CTCAE contains 124 items that measure 78 symptomatic AEs drawn from the more than 800 CTCAE items [7]. Research has shown that the English version of the PRO-CTCAE has good psychometric properties [9], and it has been translated into more than 30 languages, including the 2019 simplified Chinese version, which was translated and linguistically validated through cognitive interviewing [10–12]. However, the psychometric properties of the simplified Chinese version of the PRO-CTCAE have not been assessed.

It is essential to know the psychometric properties of new clinical assessment tools to capture the latent phenomenon it is intended to measure. To limit the burden of participants, 38 items representing 22 symptomatic toxicities were regarded as “core item set” and selected for validation. And the aim of this study was to determine the validity, reliability, and responsiveness of the simplified Chinese version of the PRO-CTCAE in order to encourage the application of the PRO-CTCAE in clinical cancer trials in Mainland China.

## Methods

### Patients

Patients who were beginning or undergoing cancer treatment at four centers in China were recruited to participate in this study between September 2019 and January 2020. The centers included the Sun Yat­sen University Cancer Center in Guangzhou, China (*n* = 1015), the Affiliated Hospital of Qingdao University in Shandong, China (*n* = 190), the Jinan Seventh People’s Hospital in Shandong, China (*n* = 240), and the First People’s Hospital of Tianmen in Hubei Province, China (*n* = 135). All the participants could speak, read, write, and understand Chinese and were 18 years of age or older.

There were no eligibility restrictions regarding disease site, current type of treatment, or type of clinical setting (inpatient or outpatient). Patients were excluded if they had a psychiatric disorder or cognitive impairment. The clinical characteristics that were collected included gender, age, education, ethnicity, marital status, diagnosis, type of treatment, and Eastern Cooperative Oncology Group Performance Status (ECOG PS). The Institutional Review Board at each site approved the study, and all the eligible patients provided informed consent.

### Measures

#### PRO-CTCAE

To reduce the burden of participants, 38 items included in the original simplified Chinese version of PRO-CTCAE library, representing 22 symptomatic toxicities, were regarded as “core item set” and selected for validation (shown in Additional file 1: Table S1). They were selected because of their prevalence in different anti-cancer treatment modalities, using surveys created by the PRO-CTCAE researchers that assessed disease sites and a set of 12 “core” symptomatic AEs recommended by the NCI, in consultation with experts in the field [13, 14]. The PRO-CTCAE evaluates the patient’s experience of each AE during the past 7 days on one to three attributes. Please visit http://healthcaredelivery.cancer.gov/pro-ctcae/ for more information about the PRO-CTCAE and authorization to use it.

#### Anchor

An anchor instrument should be used as a measurable criterion to evaluate the validity of a new scale. This study used the European Organization for Research and Treatment of Cancer Quality of Life Questionnaire-C30 (EORTC QLQ-C30) to evaluate the validity of the Chinese version of the PRO-CTCAE, based on expert advice and literature reviews. EORTC QLQ-C30 has been widely used as a gold standard in the validation and reliability of various language versions of PRO-CTCAE [9, 15–17]. The Chinese version of the EORTC QLQ-C30 was validated in Chinese patients who had cancers and was found to have good psychometric properties [18]. The EORTC QLQ-C30 contains 30 items that are divided into five functional subscales, nine symptom subscales, a global health/quality of life (QOL) subscale, and financial difficulty. The reference period is the past 7 days.

### Study design

All eligible patients who were beginning or undergoing cancer therapies were given relevant information by the site staff before they provided informed consent to participate. Participants were assigned to cohorts with different questionnaire schedules depending upon their treatment schedules, to minimize additional clinical visits. Cohort A was asked to finish both the PRO-CTCAE and EORCT-QLQ C30 during Visit 1 to analyze the validity and internal consistency of the PRO-CTCAE. Patients in Cohort B who had a planned visit in Cohort A and also had a return visit one day after their first visit (Visit 1b) were asked to complete the PRO-CTCAE a second time to analyze its test-retest reliability. Cohort C, who were in Cohort A but still had another visit within 1–2 weeks after the first visit (Visit 2) were asked to complete the PRO-CTCAE again in order to run responsiveness analyses. All the questionnaires were completed independently by the participants using paper and pencil. Site staff checked the scales after patients submitted them. If a scale had missing data, the scale was immediately given back to the participant to complete it. All scores on the PRO-CTCAE items and the EORTC QLQ-C30 subscales were transformed into a 0–100 scale, with greater function scores and global health scores indicating better QOL, and greater symptom scores indicating worse symptoms [19].

### Statistical analyses

All the statistical analyses were conducted using SPSS 26.0 and Amos 22.0 statistical software program (IBM, USA). A *p* < 0.05 was set as the criterion for statistical significance.

#### Analysis of validity

Validity refers to the ability of a scale to measure what it is designed to measure. Convergent validity, which assumes conceptually related items on a new instrument and an established instrument should have substantially high correlations, was evaluated using Pearson’s correlations between parallel items on the PRO-CTCAE and QLQ-C30. Correlation coefficients of 0.1, 0.3, and 0.5, respectively, are considered to be small, medium, and large correlations [20]. Construct validity was also estimated with confirmatory factor analysis (CFA) using structural equation modeling (SEM). The CFA model fit was assessed by the following goodness-of-fit indices: the Root Mean Square Error of Approximation (RMSEA) and the 90% RMSEA confidence interval (CI); the Comparative Fit Index (CFI); and the Incremental Fit Index (IFI). A RMSEA of ≤0.05 and ≤ 0.08 indicate good and acceptable fit, respectively, and a CFI > 0.9, and IFI > 0.9 indicate acceptable fit [21, 22]. Besides, convergent validity was evaluated by the averaged variance extracted (AVE), where an AVE > 0.5 was considered acceptable. Composite reliability (CR) values > 0.7 indicated good reliability [23]. Discriminant validity was assessed using Pearson’s correlations between various domains and the AVE. When the correlations between the dimensions were smaller than the square root of the AVE, it indicated good discriminant validity. The factor loadings should be larger than 0.5 [24]. Finally, known-groups validity was tested comparing the scores on the PRO-CTCAE items, that would be expected to be greater or smaller in one group of patients versus another group of patients, according to their diagnosis, treatment modality, or other clinical characteristic.

#### Analysis of reliability

Reliability refers to the internal consistency of the scores of items on a scale and the repeatability of scores from one administration of a scale to another administration of it. Internal consistency was assessed by Cronbach’s ɑ, where an ɑ should > 0.8. Test-retest reliability was evaluated by the intraclass correlation coefficient (ICC) using a two-way random model. The ICC is considered high when it is 0.7 or greater [25].

#### Analysis of responsiveness

Responsiveness refers to the ability to change responding to an effective intervention, which was evaluated by testing (paired t-tests) the difference between PRO-CTCAE items on the first visit (pre-treatment) and second visit (post-treatment) [26]. The duration applied to examine the responsiveness of the PRO-CTCAE was within 1–2 weeks, which was determined based on the literature [9], experience and expectation that symptomatic AEs would change after anti-tumor treatment. According to expert consultation and patient’s chief complaint, significant changes were observed in most AEs within 1–2 weeks after chemotherapy or radiotherapy. Standardized response means (SRMs), which were used as responsiveness indices, were computed by dividing the absolute difference between the mean scores by the standard deviation. Values of SRM equal to 0.20, 0.50, and 0.80, respectively are considered small, medium, and large responsiveness [27].

## Results

### Participants

**Fig. 1:**
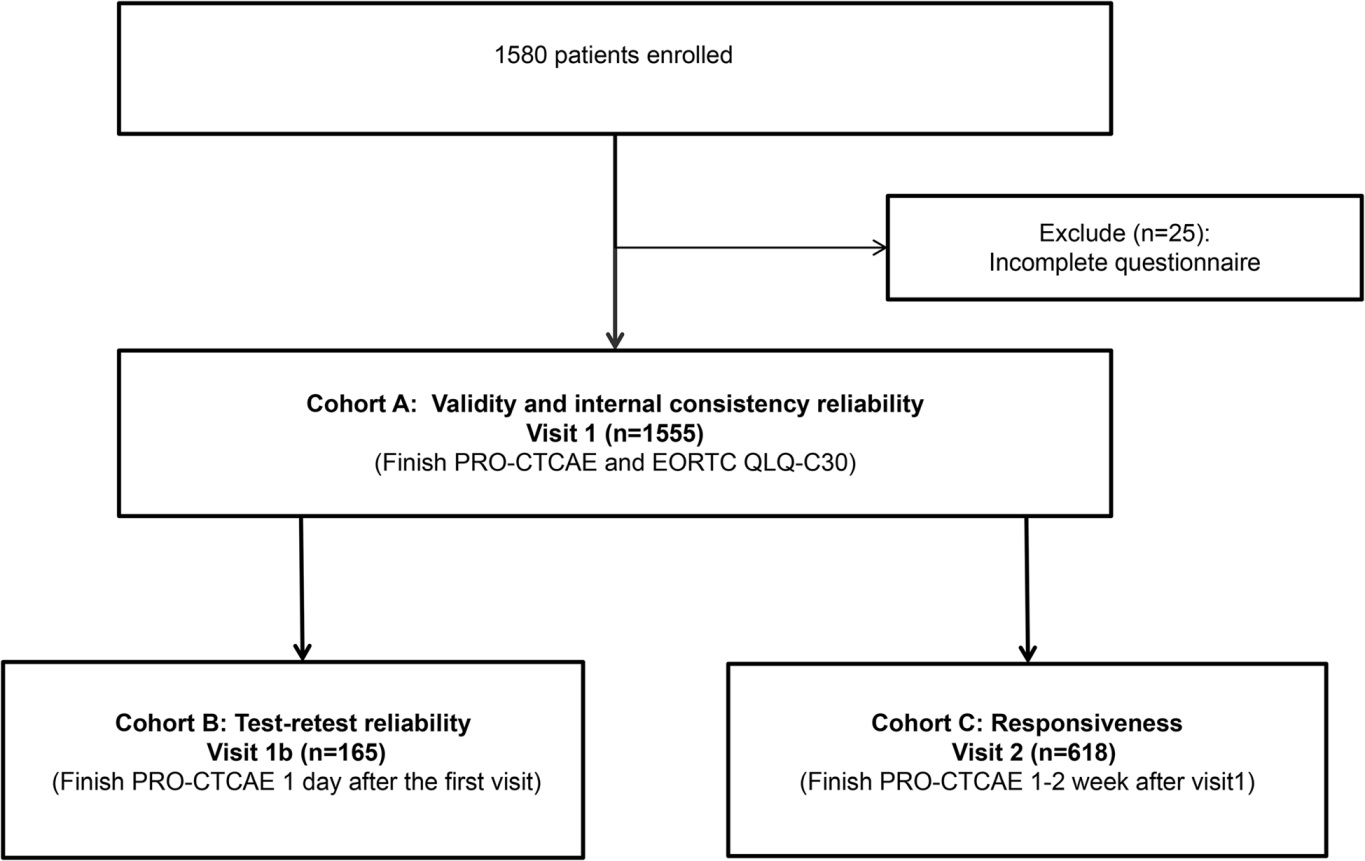
Flowchart of the included patients*.* Abbreviations: EORTC QLQ-C30, European Organisation for Research and Treatment of Cancer Quality of Life Questionnaire; PRO-CTCAE, patient-reported outcomes version of the common terminology criteria for adverse events

A total of 1580 eligible patients who were undergoing or initiating cancer therapies were enrolled in the study between September 2019 and January 2020. Of these, 98.42% completed both the PRO-CTCAE and the EORTC QLQ-C30 during Visit 1, for a total sample of 1555 patients (see Fig. 1). The characteristics of the 1555 respondents are summarized in Table 1. Their median age was 51 (range = 18–80) years, 62.4% were male, 20.1% had ECOG PS 2–4, and 48.7% received concurrent chemoradiotherapy.

**Table 1:** Patient Characteristics (*N* = 1555)

Characteristic	No. of patients	%
Age median (range), y	51 (18–80)	
Age groups		
<30	92	5.92
30–60	1090	70.10
>60	373	23.99
Gender		
Female	585	37.62
Male	970	62.38
Ethnic groups		
Han	1535	98.71
Others	20	1.29
Marital status		
Married	1296	83.34
Others	259	16.66
Education		
Primary school	292	18.78
Middle school	612	39.36
High school	353	22.70
College or more	298	19.16
Diagnosis		
NPC or HNC	871	56.01
Lung, breast	234	15.05
Gastrointestinal	214	13.76
Genitourinary or gynecologic	202	12.99
Others	34	2.19
ECOG PS at visit 1		
0–1	1243	79.94
2–4	312	20.06
Treatments		
Chemotherapy	473	30.42
Chemotherapy + radiotherapy	757	48.68
Radiotherapy	325	20.90

Abbreviations: ECOG PS, Eastern Cooperative Oncology Group Performance Status; HNC, head and neck cancer; NPC, nasopharyngeal carcinoma

**Fig. 2:**
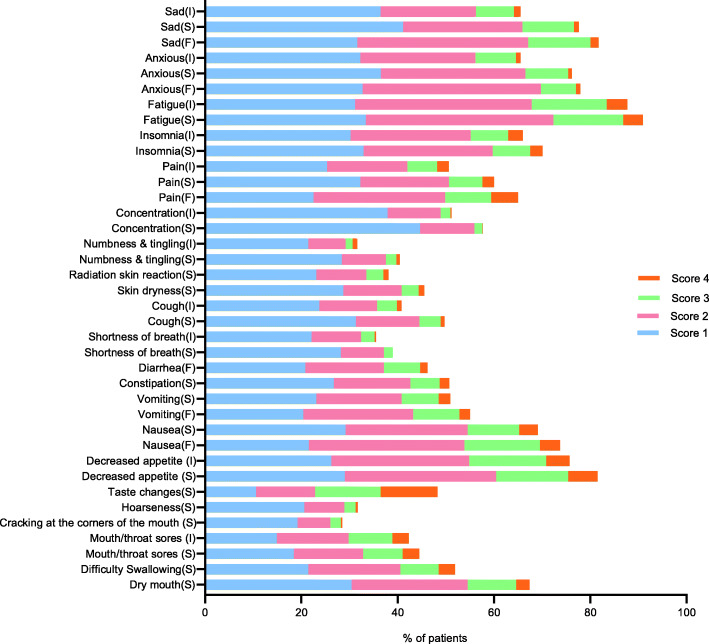
Distribution of PRO-CTCAE item scores at Visit 1. Frequency: 0 = “Never”, 1 = “Rarely”, 2 = “Occasionally”, 3 = “Frequently”, 4 = “Almost constantly”. Interference: 0=“Not at all”, 1=“A little bit”, 2 = “Somewhat”, 3=“Quite a bit”, 4 = “Very much”. Severity: 0 = “None”, 1 = “Mild”, 2 = “Moderate”, 3 = “Severe”, 4 = “Very severe”. Abbreviations: F, Frequency; I, Interference with daily activities; PRO-CTCAE, patient-reported outcomes version of the common terminology criteria for adverse events; S, Severity

During their first visit, nearly all the patients (99.4%) had a score greater than 0 on at least one item, and 92.54% had a score of 2. Extensive symptoms were reported, with the occurrence of a median (range) of 19 (0–38) symptoms. The detailed distribution of item scores is presented in Fig. 2.

### Validity

#### Construct validity

**Fig. 3:**
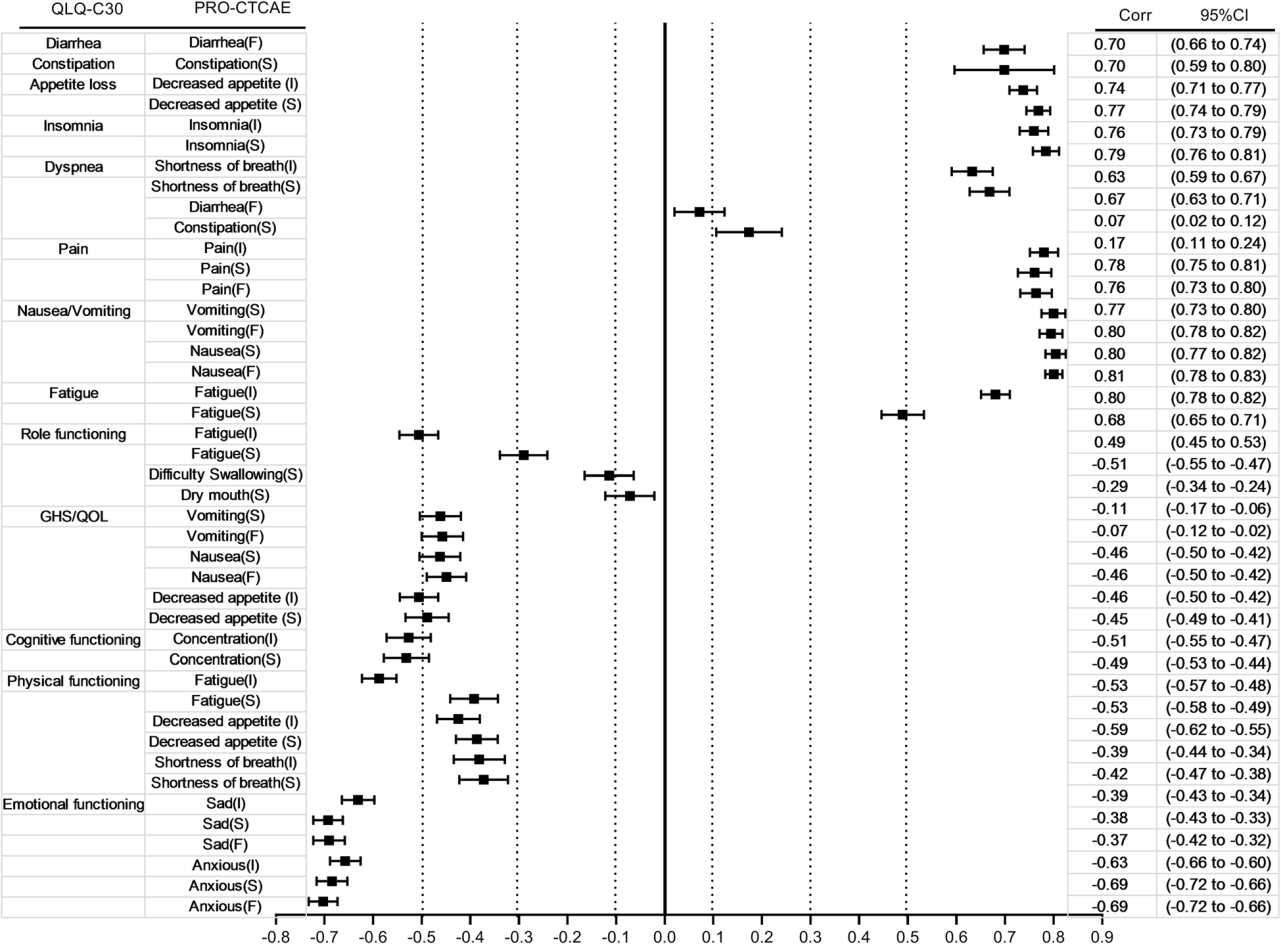
Pearson’s correlations between PRO-CTCAE items and QLQ-C30 domains at Visit 1. Abbreviations: CI, Confidence interval; Corr, correlation; F, Frequency; I, Interference with daily activities; PRO-CTCAE, patient-reported outcomes version of the common terminology criteria for adverse events; QLQ-C30, quality of life questionnaire C30; S, Severity

The Pearson’s correlations of the PRO-CTCAE items and QLQ-C30 domain are presented in Fig. 3. All of the PRO-CTCAE items were significantly related to, and in the expected direction with the QLQ-C30 domains (all *p* < 0.05). The PRO-CTCAE symptoms that are likely to affect certain types of functioning tended to be strongly associated with the conceptually related QLQ-C30 functioning scales. For example, the PRO-CTCAE items describing anxiety and sadness had the highest correlations with the QLQ-C30 emotional functioning scale (Pearson’s ∣*r*∣ > 0.60, *p* < 0.001) (Additional file 1: Table S2). In addition, the similar symptom items/scales between the PRO-CTCAE and the QLQ-C30 had large correlations, with all the *r* > 0.60, except for fatigue (severity: *r* = 0.49, 95% CI: 0.45–0.53, *p* < 0.001) (Additional file 1: Table S3). Correspondingly, the correlations of conceptually related items between the two scales were much larger than the correlations of the unrelated items, which indicated good item convergent validity.

CFA showed that the hypothesized model had favorable goodness-of-fit indices: RMSEA = 0.046 (90% CI = 0.044–0.048), χ^2^/df = 4.327, IFI = 0.964, and CFI = 0.964. The results for convergent validity are presented in Table 2. The factor loadings of the items were higher than 0.65, the CR value for each subscale was more than 0.90, and the AVE values ranged from 0.58 to 0.90. The correlation matrix of the domains in the analysis of discriminant validity are shown in Additional file 1: Table S4. The correlations between the dimensions varied between 0.02 and 0.61, which were smaller than the square root of the AVE. These CFA results indicated that all the domains had satisfactory convergent and discriminant validity.

**Table 2:** Results of the structural validity analysis (*N* = 1555)

Components	Item	Factor loadings	AVE	CR
Radiation reaction	Dry mouth(S)	0.77	0.58	0.93
	Difficulty Swallowing(S)	0.85		
	Mouth/throat sores (S)	0.88		
	Mouth/throat sores (I)	0.86		
	Cracking at the corners of the mouth (S)	0.72		
	Hoarseness(S)	0.65		
	Taste changes(S)	0.77		
	Skin dryness(S)	0.67		
	Radiation skin reaction(S)	0.68		
Anxiety and sadness	Anxious(F)	0.83	0.77	0.95
	Anxious(S)	0.86		
	Anxious(I)	0.90		
	Sad(F)	0.87		
	Sad(S)	0.94		
	Sad(I)	0.87		
Nausea and vomiting	Nausea(F)	0.90	0.72	0.91
	Nausea(S)	0.98		
	Vomiting(F)	0.73		
	Vomiting(S)	0.76		
Pain	Pain(F)	0.88	0.85	0.94
	Pain(S)	0.98		
	Pain(I)	0.90		
Fatigue	Fatigue(S)	0.94	0.92	0.96
	Fatigue(I)	0.98		
Decreased appetite	Decreased appetite (S)	0.94	0.88	0.94
	Decreased appetite (I)	0.94		
Concentration	Concentration(S)	0.92	0.83	0.91
	Concentration(I)	0.90		
Numbness and tingling	Numbness & tingling(S)	0.88	0.83	0.90
	Numbness & tingling(I)	0.93		
Insomnia	Insomnia(S)	0.91	0.86	0.93
	Insomnia(I)	0.95		
Cough	Cough(S)	0.93	0.86	0.93
	Cough(I)	0.93		
Dyspnoea	Shortness of breath(S)	0.93	0.90	0.95
	Shortness of breath(I)	0.97		

Abbreviations: AVE, Average Variance Extracted; CR, Construct Reliability; F, Frequency; I, Interference with daily activities; S, Severity

#### Known-groups validity

**Fig. 4:**
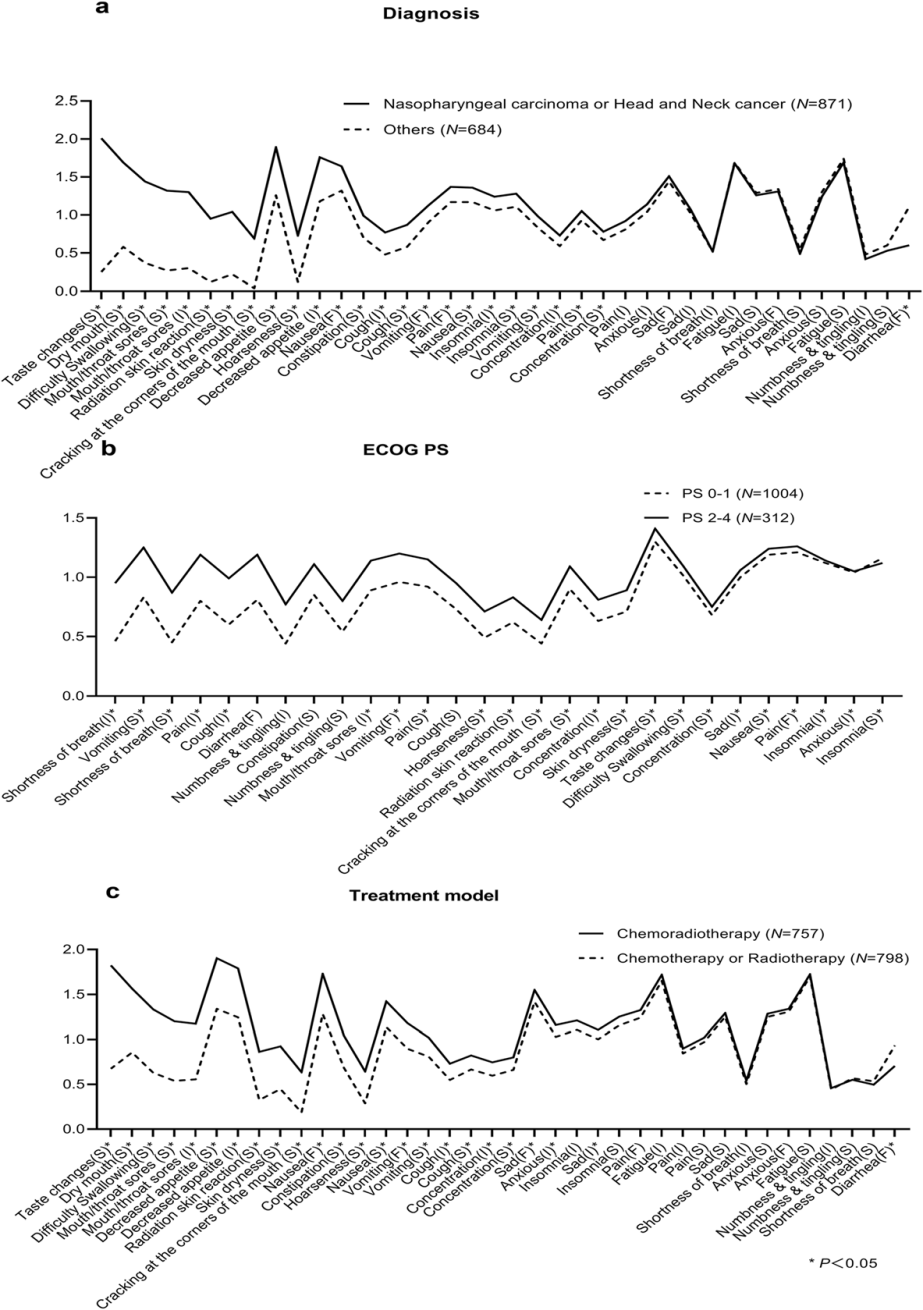
Known groups validity for diagnosis (**A**), ECOG PS (**B**), and treatment (**C**). Abbreviations: ECOG PS, Eastern Cooperative Oncology Group Performance Status; F, Frequency; I, Interference with daily activities; S, Severity

The analysis of known-groups validity used *t*-tests to compare the PRO-CTCAE scores based on diagnosis, PS, and treatment. As shown in Fig. 4, a majority of PRO-CTCAE items (24 of 38 items), especially radiotherapy-related items, were observed higher scores in patients with nasopharyngeal carcinoma (NPC) or head and neck cancer (HNC) compared with those of cancers from other sites (*p* < 0.05). Compared with patients received concurrent chemoradiotherapy, patients accepted single treatment modality had lower mean item scores (23 of 38 items, p < 0.05). And patients with PS 2–4 had higher scores than those with PS 0–1 in 22 PRO-CTCAE items, such as pain (severity and interference, *p* < 0.001).

### Reliability

As shown in Additional file 1: Table S5, the Cronbach’s ɑ of all item clusters on the scale was greater than 0.9. The test-retest reliability results for the 165 participants who completed the PRO-CTCAE twice (one day apart) are presented in Table 3. The median ICC of 38 items on the scale was 0.85 (range = 0.71–0.91). Thus, the Cronbach’s ɑs and ICCs indicated that the scale had good reliability.

**Table 3:** Test-retest reliability of the Chinese PRO-CTCAE (*N* = 165)

Item	Intra-class Correlation Coefficient		
	Frequency	Severity	Interference
Dry mouth	–	0.86	–
Difficulty Swallowing	–	0.89	–
Mouth/throat sores	–	0.86	0.87
Cracking at the corners of the mouth	–	0.85	–
Hoarseness	–	0.84	–
Taste changes	–	0.88	–
Decreased appetite	–	0.85	0.89
Nausea	0.91	0.89	–
Vomiting	0.9	0.90	–
Constipation	–	0.84	–
Diarrhea	0.80		–
Shortness of breath	–	0.74	0.71
Cough	–	0.85	0.90
Skin dryness	–	0.84	–
Radiation skin reaction	–	0.88	–
Numbness & tingling	–	0.85	0.82
Concentration	–	0.73	0.74
Pain	0.88	0.72	0.80
Insomnia	–	0.82	0.84
Fatigue	–	0.83	0.84
Anxious	0.88	0.83	0.83
Sad	0.87	0.88	0.84

Abbreviations: PRO-CTCAE, patient-reported outcomes version of the common terminology criteria for adverse events

### Responsiveness

Based on questionnaires of 618 patients, the mean change in PRO-CTCAE scores was statistically significant, increasing for 25 of the 38 items (*p* < 0.05), and decreasing for 2 of the 38 items: hoarseness (S) (*p* < 0.001) and diarrhea (F) (*p* = 0.042). The SRMs of 14 PRO-CTCAE items were greater than 0.8, indicating strong responsiveness. On the other hand, some items had small SRM values (< 0.2), reflecting weak responsiveness, e.g., concentration, anxiety (“anxious”), and sadness (“sad”) (see Additional file 1: Table S6).

## Discussion

This large-scale multicenter study to assess the core item set of the simplified Chinese language PRO-CTCAE demonstrated that it generally had excellent psychometric properties with respect to reliability, validity, and responsiveness. This is the first study that has quantitatively evaluated this core item set of the simplified Chinese version of the scale, and our findings support the incorporation of it into AE reporting in future cancer clinical trials.

The assessment of validity, which should include convergent and discriminant validity, is critically important for evaluating a new PRO measure [28]. To establish convergent validity, the analyses should demonstrate that measures that are presumed to be related are actually related; to establish discriminant validity, the analyses should demonstrate that measures that are presumed to be unrelated are actually unrelated. The current study’s correlation analysis indicated that the scale’s convergent validity was good, with most of the parallel items of the PRO-CTCAE and QLQ-C30 scales being highly correlated. An exception to these findings was that large correlations were observed between decreased appetite and items that were not conceptually related, including nausea/vomiting and fatigue. These findings may be explained by the fact that patients with cancer typically experience multiple symptoms, and one of these symptoms might trigger the other symptoms in a cluster [29]. While QLQ-C30 items reflect the frequency of a symptom, PRO-CTCAE evaluates the patient’s experience of each AE on one to three attributes of frequency, severity, interference, amount, presence / absence. Thus, the severity of fatigue in PRO-CTCAE may have relatively small correlations with frequency of fatigue in QLQ C-30. Subsequently, CFA model was used to assess the construct validity. The results of CFA also indicated that all the domains had satisfactory convergent and discriminant validity. CFA demonstrated that the dimensions of the scale were consistent with their original concepts, and the structural model had an excellent fit, which confirmed the construct validity of the PRO-CTCAE.

In addition, the results of known-group validity (or clinical validity) showed that the scores on the PRO-CTCAE items differed significantly based on different types of cancer, PS scores, and treatment groups. Patients diagnosed with NPC or HNC tended to have higher PRO-CTCAE scores, especially on items related to radiation reactions, since most patients with NPC or HNC receive radiotherapy. However, the scores of emotion-associated items, such as anxiety, did not differ significantly across different groups, probably because of the variety and complexity of emotional experiences [30].

Responsiveness is divided into internal and external responsiveness [26], and our analysis of internal responsiveness showed that those PRO-CTCAE items reflecting acute AEs had larger SRMs, while other items had smaller SRMs. Some PRO-CTCAE items, such as concentration, had stable scores, and the interval we used (1–2 weeks) may have been too short to make significant change in scores.

Similar to our results, studies of other language versions of the PRO-CTCAE have demonstrated that it has good measurement properties [9, 15–17]. For example, validation of the original English version of PRO-CTCAE showed good test-retest reliability, with an ICC of 0.76, and good validity, with strong correlations between related PRO-CTCAE items and QLQ-C30 domains, as well as significant changes after treatment [9]. In addition, Hagelstein et al., who studied a core item set of the German version of the PRO-CTCAE, reported that the Cronbach’s ɑ of seven of the nine item clusters was above 0.9, and the version’s validity was demonstrated by PCA, Multitrait-multimethod matrix (MTMM), and known groups analyses [15].

The current study included a diverse sample, which helps make our results generalizable. First, a wide range of types of cancer, including less common ones, enhanced the diversity of the sample. Second, the proportion of patients in the sample with poor PS (ECOG PS ≥2) makes our findings on the simple Chinese PRO CTCAE applicable to patients with severe symptoms. Moreover, the centers involved in the study were located across China, including Northern China and Southern China and urban and rural areas, reflecting a diversity of geography, race, education, and economic circumstances.

We acknowledge that there are some limitations with the current study. Considering patient compliance, we did not include all PRO-CTCAE items for validation, which was consistent with validation of PRO-CTCAE in other languages [9, 15, 16]. To minimize respondent burden and to avoid missing data, the psychometric analysis was confined to 38 selected items, and the results should not be extrapolated to the entire Chinese language item-library of the PRO-CTCAE. Thus, future studies that examine the other cancer-specific PRO-CTCAE items are warranted. All the PRO information used in this study was collected by paper and pencil. However, that acceptable equivalence has been found between the web, paper, and automated telephone interactive voice-response administration of the English version of the PRO-CTCAE [31]. Finally, because half of the patients in this study were in an endemic area for NPC, there was over-representation of patients with the nine symptoms related to “radiation reaction” in the study. A large number of cases and a wider range of types of cancer from diverse areas of China would offset this sampling bias.

In conclusion, this large-scale, multicenter study demonstrated that the simplified Chinese version of the PRO-CTCAE has good reliability, validity, and responsiveness, and that it can be used to supplement the AE reports of clinicians in cancer clinical trials in Mainland China.

## Supplementary Information

**Additional file 1 Table S1.** Item clusters of the PRO-CTCAE. **Table S2.** Criteria-related validity: Pearson’s correlation coefficients between PRO-CTCAE items and QLQ-C30 functional domains (*N* = 1555)**. Table S3.** Criteria-related validity: Pearson correlation coefficients between similar symptom items on the PRO-CTCAE and QLQ-C30 (*N* = 1555)**. Table S4.** Correlation matrix of factors (*N* = 1555)**. Table S5.** Cronbach’s alpha of each dimension of the simple Chinese PRO-CTCAE (*N* = 1555)**. Table S6.** Responsiveness of the Chinese PRO-CTCAE (*N* = 618)

## Data Availability

The data and materials are available from the corresponding author on reasonable request.

## Abbreviations

AEs: Adverse Events
AVE: Average Variance Extracted
CR: Construct Reliability
CFA: Confirmatory Factor Analysis
ECOG PS: Eastern Cooperative Oncology Group Performance Status
HNC: head and neck cancer
ICC: Intraclass Correlation
NPC: Nasopharyngeal Carcinoma
PRO-CTCAE: patient-reported outcomes version of the common terminology criteria for adverse events
QLQ: Quality of Life Questionnaire
SRMs: Standardized Response Means.
