# Supplementary material for: Validity and reliability of the simplified Chinese patient-reported outcomes version of the common terminology criteria for adverse events
Source: BMC Cancer. 2021 Jul 27;21:860. doi: 10.1186/s12885-021-08610-0 (PMC8314582; doi:10.1186/s12885-021-08610-0)
Supplement: Supplementary file 1 — Additional file 1 Table S1. Item clusters of the PRO-CTCAE. Table S2. Criteria-related validity: Pearson’s correlation coefficients between PRO-CTCAE items and QLQ-C30 functional domains (N = 1555). Table S3. Criteria-related validity: Pearson correlation coefficients between similar symptom items on the PRO-CTCAE and QLQ-C30 (N = 1555). Table S4. Correlation matrix of factors (N = 1555). Table S5. Cronbach’s alpha of each dimension of the simple Chinese PRO-CTCAE (N = 1555). Table S6. Responsiveness of the Chinese PRO-CTCAE (N = 618) [file 12885_2021_8610_MOESM1_ESM.doc]

**Additional file 1**

**Table S1.** Item clusters of the PRO-CTCAE.

**Table S2.** Criteria-related validity: Pearson’s correlation coefficients between PRO-CTCAE items and QLQ-C30 functional domains (*N*=1555)

**Table S3.** Criteria-related validity: Pearson correlation coefficients between similar symptom items on the PRO-CTCAE and QLQ-C30 (*N*=1555)

**Table S4.** Correlation matrix of factors (*N*=1555)

**Table S5.** Cronbach’s alpha of each dimension of the simple Chinese PRO-CTCAE (*N*=1555)

**Table S6.** Responsiveness of the Chinese PRO-CTCAE (*N*=618)

**Table S1.** Item clusters of the PRO-CTCAE

| Item cluster | Number of items | Item | Item dimensions |
| --- | --- | --- | --- |
| Radiation reaction | 9 | Dry mouth(S) | Severity, interference |
|  |  | Difficulty Swallowing(S) |  |
|  |  | Mouth/throat sores (S) |  |
|  |  | Mouth/throat sores (I) |  |
|  |  | Cracking at the corners of the mouth (S) |  |
|  |  | Hoarseness(S) |  |
|  |  | Taste changes(S) |  |
|  |  | Skin dryness(S) |  |
|  |  | Radiation skin reaction(S) |  |
| Anxiety and sadness | 6 | Anxious(F) | Frequency, severity |
|  |  | Anxious(S) | interference |
|  |  | Anxious(I) |  |
|  |  | Sad(F) |  |
|  |  | Sad(S) |  |
|  |  | Sad(I) |  |
| Nausea and vomiting | 4 | Nausea(F) | Frequency, severity |
|  |  | Nausea(S) |  |
|  |  | Vomiting(F) |  |
|  |  | Vomiting(S) |  |
| Pain | 3 | Pain(F) | Frequency, severity |
|  |  | Pain(S) | interference |
|  |  | Pain(I) |  |
| Fatigue | 2 | Fatigue(S) | Severity, interference |
|  |  | Fatigue(I) |  |
| Decreased appetite | 2 | Decreased appetite (S) | Severity, interference |
|  |  | Decreased appetite (I) |  |
| Concentration | 2 | Concentration(S) | Severity, interference |
|  |  | Concentration(I) |  |
| Numbness and tingling | 2 | Numbness & tingling(S) | Severity, interference |
|  |  | Numbness & tingling(I) |  |
| Insomnia | 2 | Insomnia(S) | Severity, interference |
|  |  | Insomnia(I) |  |
| Cough | 2 | Cough(S) | Severity, interference |
|  |  | Cough(I) |  |
| Dyspnoea | 2 | Shortness of breath(S) | Severity, interference |
|  |  | Shortness of breath(I) |  |
| Diarrhea | 1 | Diarrhea(F) | Frequency |
| Constipation | 1 | Constipation(S) | Severity |
| Abbreviations: F, Frequency; I, Interference with daily activities; PRO-CTCAE, patient-reported outcomes version of the common terminology criteria for adverse events; S, Severity | | | |

**Table S2.** Criteria-related validity: Pearson’s correlation coefficients between PRO-CTCAE items and QLQ-C30 functional domains (*N*=1555)

|  | QLQ-C30 domains | | | | | | |
| --- | --- | --- | --- | --- | --- | --- | --- |
| PRO-CTCAE items | Physical functioning | | Role functioning | | Emotional functioning | Cognitive functioning | GHS/QOL |
| Dry mouth(S) | -0.15 | -0.07 | | -0.11 | | -0.15 | -0.26 |
| Difficulty Swallowing(S) | -0.18 | -0.11 | | -0.16 | | -0.17 | -0.30 |
| Mouth/throat sores (S) | -0.17 | -0.15 | | -0.14 | | -0.15 | -0.30 |
| Mouth/throat sores (I) | -0.19 | -0.18 | | -0.15 | | -0.17 | -0.31 |
| Cracking at the corners of the mouth (S) | -0.13 | -0.06 | | -0.08 | | -0.17 | -0.23 |
| Hoarseness(S) | -0.13 | -0.11 | | -0.10 | | -0.16 | -0.24 |
| Taste changes(S) | -0.12 | -0.12 | | -0.13 | | -0.11 | -0.26 |
| Decreased appetite (S) | -0.39 | -0.34 | | -0.31 | | -0.26 | -0.49 |
| Decreased appetite (I) | -0.42 | -0.40 | | -0.35 | | -0.30 | -0.51 |
| Nausea(F) | -0.34 | -0.27 | | -0.25 | | -0.29 | -0.45 |
| Nausea(S) | -0.38 | -0.33 | | -0.28 | | -0.27 | -0.46 |
| Vomiting(F) | -0.32 | -0.26 | | -0.19 | | -0.28 | -0.46 |
| Vomiting(S) | -0.35 | -0.29 | | -0.23 | | -0.27 | -0.46 |
| Constipation(S) | -0.20 | -0.15 | | -0.09 | | -0.21 | -0.29 |
| Diarrhea(F) | -0.12 | -0.05 | | -0.11 | | -0.22 | -0.16 |
| Shortness of breath(S) | -0.37 | -0.28 | | -0.28 | | -0.36 | -0.36 |
| Shortness of breath(I) | -0.38 | -0.30 | | -0.27 | | -0.35 | -0.37 |
| Cough(S) | -0.20 | -0.12 | | -0.16 | | -0.18 | -0.28 |
| Cough(I) | -0.25 | -0.18 | | -0.21 | | -0.23 | -0.32 |
| Skin dryness(S) | -0.17 | -0.11 | | -0.12 | | -0.17 | -0.26 |
| Radiation skin reaction(S) | -0.11 | -0.15 | | -0.06 | | -0.09 | -0.04 |
| Numbness & tingling(S) | -0.29 | -0.13 | | -0.13 | | -0.26 | -0.23 |
| Numbness & tingling(I) | -0.33 | -0.15 | | -0.20 | | -0.28 | -0.27 |
| Concentration(S) | -0.40 | -0.33 | | -0.42 | | -0.53 | -0.39 |
| Concentration(I) | -0.40 | -0.33 | | -0.39 | | -0.53 | -0.36 |
| Pain(F) | -0.31 | -0.26 | | -0.30 | | -0.24 | -0.33 |
| Pain(S) | -0.32 | -0.28 | | -0.31 | | -0.23 | -0.35 |
| Pain(I) | -0.36 | -0.29 | | -0.33 | | -0.28 | -0.38 |
| Insomnia(S) | -0.31 | -0.23 | | -0.33 | | -0.34 | -0.33 |
| Insomnia(I) | -0.33 | -0.24 | | -0.35 | | -0.36 | -0.33 |
| Fatigue(S) | -0.39 | -0.29 | | -0.35 | | -0.34 | -0.38 |
| Fatigue(I) | -0.59 | -0.51 | | -0.44 | | -0.41 | -0.49 |
| Anxious(F) | -0.40 | -0.40 | | -0.70 | | -0.38 | -0.31 |
| Anxious(S) | -0.40 | -0.40 | | -0.69 | | -0.38 | -0.33 |
| Anxious(I) | -0.39 | -0.38 | | -0.66 | | -0.43 | -0.37 |
| Sad(F) | -0.40 | -0.38 | | -0.69 | | -0.40 | -0.36 |
| Sad(S) | -0.40 | -0.41 | | -0.69 | | -0.37 | -0.33 |
| Sad(I) | -0.36 | -0.36 | | -0.63 | | -0.40 | -0.35 |
| Abbreviations: F, Frequency; GHS/QOL, global health/quality of life; I, Interference with daily activities; PRO-CTCAE, patient-reported outcomes version of the common terminology criteria for adverse events; QLQ-C30, quality of life questionnaire C30; S, Severity; | | | | | | | |

**Table S3.** Criteria-related validity: Pearson correlation coefficients between similar symptom items on the PRO-CTCAE and QLQ-C30 *(N*=1555)

|  | QLQ-C30 domains | | | | | | | |
| --- | --- | --- | --- | --- | --- | --- | --- | --- |
| PRO-CTCAE  items | Fatigue | Nausea/  Vomiting | Pain | Dyspnea | Insomnia | Appetite loss | Constipation | Diarrhea |
| Fatigue(S) | 0.49 | 0.34 | 0.32 | 0.19 | 0.55 | 0.44 | 0.23 | 0.11 |
| Fatigue(I) | 0.68 | 0.41 | 0.34 | 0.31 | 0.35 | 0.49 | 0.21 | 0.14 |
| Nausea(F) | 0.39 | 0.80 | 0.21 | 0.22 | 0.22 | 0.51 | 0.25 | 0.10 |
| Nausea(S) | 0.41 | 0.81 | 0.24 | 0.23 | 0.22 | 0.52 | 0.24 | 0.07 |
| Vomiting(F) | 0.32 | 0.80 | 0.21 | 0.20 | 0.18 | 0.42 | 0.26 | 0.10 |
| Vomiting(S) | 0.33 | 0.80 | 0.24 | 0.21 | 0.17 | 0.42 | 0.27 | 0.09 |
| Pain(F) | 0.31 | 0.24 | 0.77 | 0.16 | 0.26 | 0.32 | 0.18 | 0.12 |
| Pain(S) | 0.33 | 0.24 | 0.76 | 0.17 | 0.27 | 0.32 | 0.17 | 0.09 |
| Pain(I) | 0.37 | 0.24 | 0.78 | 0.18 | 0.28 | 0.33 | 0.17 | 0.11 |
| Shortness of breath(S) | 0.32 | 0.27 | 0.23 | 0.67 | 0.25 | 0.25 | 0.23 | 0.14 |
| Shortness of breath(I) | 0.34 | 0.28 | 0.25 | 0.63 | 0.24 | 0.26 | 0.23 | 0.13 |
| Insomnia(S) | 0.38 | 0.26 | 0.34 | 0.21 | 0.79 | 0.29 | 0.16 | 0.18 |
| Insomnia(I) | 0.40 | 0.27 | 0.35 | 0.22 | 0.76 | 0.30 | 0.18 | 0.19 |
| Decreased appetite (S) | 0.49 | 0.52 | 0.31 | 0.24 | 0.26 | 0.77 | 0.27 | 0.04 |
| Decreased appetite (I) | 0.52 | 0.52 | 0.35 | 0.25 | 0.29 | 0.74 | 0.28 | 0.06 |
| Constipation(S) | 0.26 | 0.25 | 0.20 | 0.17 | 0.19 | 0.31 | 0.70 | 0.03 |
| Diarrhea(F) | 0.11 | 0.09 | 0.12 | 0.07 | 0.12 | 0.05 | 0.04 | 0.70 |
| Abbreviations: F, Frequency; I, Interference with daily activities; PRO-CTCAE, patient-reported outcomes version of the common terminology criteria for adverse events; QLQ-C30, quality of life questionnaire C30; S, Severity | | | | | | | | |

**Table S4.** Correlation matrix of factors (*N*=1555)

| Dimensions | Cough | Insomnia | Numbness and tingling | Concentration | Decreased appetite | Fatigue | Pain | Nausea and vomiting | Anxiety and sadness | Radiation reaction | Dyspnoea | Diarrhea | Constipation |
| --- | --- | --- | --- | --- | --- | --- | --- | --- | --- | --- | --- | --- | --- |
| Cough | 0.93 |  |  |  |  |  |  |  |  |  |  |  |  |
| Insomnia | 0.39 | 0.93 |  |  |  |  |  |  |  |  |  |  |  |
| Numbness and tingling | 0.33 | 0.20 | 0.91 |  |  |  |  |  |  |  |  |  |  |
| Concentration | 0.40 | 0.28 | 0.35 | 0.91 |  |  |  |  |  |  |  |  |  |
| Decreased appetite | 0.34 | 0.37 | 0.22 | 0.41 | 0.94 |  |  |  |  |  |  |  |  |
| Fatigue | 0.38 | 0.28 | 0.26 | 0.51 | 0.57 | 0.96 |  |  |  |  |  |  |  |
| Pain | 0.24 | 0.27 | 0.33 | 0.31 | 0.37 | 0.37 | 0.92 |  |  |  |  |  |  |
| Nausea and vomiting | 0.31 | 0.27 | 0.21 | 0.34 | 0.61 | 0.44 | 0.26 | 0.85 |  |  |  |  |  |
| Anxiety and sadness | 0.32 | 0.23 | 0.20 | 0.49 | 0.41 | 0.55 | 0.38 | 0.35 | 0.88 |  |  |  |  |
| Radiation reaction | 0.30 | 0.49 | 0.20 | 0.29 | 0.61 | 0.31 | 0.38 | 0.32 | 0.21 | 0.76 |  |  |  |
| Dyspnoea | 0.30 | 0.28 | 0.25 | 0.41 | 0.36 | 0.47 | 0.35 | 0.27 | 0.41 | 0.34 | 0.95 |  |  |
| Diarrhea | 0.16 | 0.00 | 0.27 | 0.17 | 0.05 | 0.10 | 0.11 | 0.08 | 0.12 | -0.07 | 0.16 | - |  |
| Constipation | 0.25 | 0.22 | 0.21 | 0.22 | 0.33 | 0.22 | 0.18 | 0.27 | 0.10 | 0.30 | 0.17 | 0.02 | - |
| Note: The diagonal values are the square root of the Average Variance Extracted (AVE) for each factor. | | | | | | | | | | | | | |

**Table S5.** Cronbach’s alpha of each dimension of the simple Chinese PRO-CTCAE (*N*=1555)

| Item cluster | Number of items | Cronbach’s ɑ | Item dimensions |
| --- | --- | --- | --- |
| Radiation reaction | 9 | 0.92 | Severity, interference |
| Anxiety and sadness | 6 | 0.95 | Frequency, severity,  interference |
| Nausea and vomiting | 4 | 0.93 | Frequency, severity |
| Pain | 3 | 0.94 | Frequency, severity,  interference |
| Fatigue | 2 | 0.96 | Severity, interference |
| Decreased appetite | 2 | 0.94 | Severity, interference |
| Numbness and tingling | 2 | 0.90 | Severity, interference |
| Dyspnoea | 2 | 0.92 | Severity, interference |
| Insomnia | 2 | 0.95 | Severity, interference |
| Cough | 2 | 0.93 | Severity, interference |
| Concentration | 2 | 0.91 | Severity, interference |
| Diarrhea | 1 | - | Frequency |
| Constipation | 1 | - | Severity |
| Totall | 38 | 0.94 |  |
| Abbreviations: PRO-CTCAE, patient-reported outcomes version of the common terminology criteria for adverse events | | | |

**Table S6.** Responsiveness of the Chinese PRO-CTCAE (*N*=618)

|  | Pre-treatment | |  | Post-treatment | |  | Differences | | SRM | t | *p* |
| --- | --- | --- | --- | --- | --- | --- | --- | --- | --- | --- | --- |
| Item | Mean | SD |  | Mean | SD |  | Mean | SD |  |  |  |
| Dry mouth(S) | 1.68 | 1.03 |  | 2.89 | 0.89 |  | -1.22 | 0.94 | 1.29 | -32.07 | ＜0.001 |
| Difficulty Swallowing(S) | 1.42 | 1.18 |  | 2.43 | 0.88 |  | -1.01 | 1.09 | 0.93 | -23.01 | ＜0.001 |
| Mouth/throat sores (S) | 1.35 | 1.23 |  | 2.54 | 0.89 |  | -1.19 | 1.11 | 1.08 | -26.77 | ＜0.001 |
| Mouth/throat sores (I) | 1.30 | 1.25 |  | 2.63 | 0.95 |  | -1.33 | 1.16 | 1.15 | -28.62 | ＜0.001 |
| Cracking at the corners of the mouth (S) | 0.70 | 0.87 |  | 1.05 | 0.85 |  | -0.35 | 0.73 | 0.48 | -12.01 | ＜0.001 |
| Hoarseness(S) | 0.77 | 0.89 |  | 0.94 | 0.87 |  | 0.10 | 0.69 | 0.15 | -6.18 | ＜0.001 |
| Taste changes(S) | 1.97 | 1.44 |  | 3.20 | 0.89 |  | -1.23 | 1.24 | 0.99 | -24.52 | ＜0.001 |
| Decreased appetite (S) | 1.83 | 1.12 |  | 2.73 | 0.87 |  | -0.90 | 0.99 | 0.91 | -22.57 | ＜0.001 |
| Decreased appetite (I) | 1.71 | 1.16 |  | 2.73 | 0.90 |  | -1.01 | 1.06 | 0.96 | -23.78 | ＜0.001 |
| Nausea(F) | 1.37 | 1.14 |  | 2.11 | 1.19 |  | -0.50 | 1.13 | 0.44 | -14.49 | ＜0.001 |
| Nausea(S) | 1.15 | 1.17 |  | 1.31 | 1.12 |  | -0.74 | 1.27 | 0.58 | -3.33 | 0.001 |
| Vomiting(F) | 0.96 | 1.11 |  | 1.25 | 1.18 |  | -0.16 | 1.20 | 0.13 | -5.66 | ＜0.001 |
| Vomiting(S) | 1.00 | 1.05 |  | 1.08 | 1.08 |  | -0.28 | 1.24 | 0.23 | -1.64 | 0.102 |
| Constipation(S) | 0.62 | 0.82 |  | 0.55 | 0.75 |  | -0.08 | 1.23 | 0.07 | 1.74 | 0.083 |
| Diarrhea(F) | 0.52 | 0.74 |  | 0.59 | 0.69 |  | 0.07 | 1.06 | 0.07 | -2.27 | 0.024 |
| Shortness of breath(S) | 0.54 | 0.84 |  | 0.56 | 0.78 |  | -0.07 | 0.76 | 0.09 | -0.33 | 0.74 |
| Shortness of breath(I) | 0.88 | 0.94 |  | 1.41 | 0.89 |  | -0.01 | 0.97 | 0.01 | -16.88 | ＜0.001 |
| Cough(S) | 0.77 | 0.97 |  | 1.41 | 0.94 |  | -0.53 | 0.79 | 0.68 | -19.65 | ＜0.001 |
| Cough(I) | 1.04 | 0.89 |  | 1.81 | 0.83 |  | -0.64 | 0.81 | 0.79 | -23.46 | ＜0.001 |
| Skin dryness(S) | 1.54 | 1.31 |  | 2.04 | 1.07 |  | -0.77 | 0.82 | 0.94 | -10.76 | ＜0.001 |
| Radiation skin reaction(S) | 0.53 | 0.80 |  | 0.56 | 0.73 |  | -0.72 | 0.81 | 0.89 | -1.30 | 0.193 |
| Numbness & tingling(S) | 0.43 | 0.77 |  | 0.44 | 0.68 |  | -0.03 | 0.59 | 0.05 | -0.36 | 0.72 |
| Numbness & tingling(I) | 0.89 | 0.98 |  | 0.86 | 0.87 |  | -0.01 | 0.67 | 0.01 | 1.11 | 0.268 |
| Concentration(S) | 0.72 | 0.80 |  | 0.74 | 0.85 |  | -0.02 | 0.72 | 0.03 | -0.48 | 0.632 |
| Concentration(I) | 1.35 | 1.17 |  | 2.50 | 0.92 |  | -0.02 | 0.92 | 0.02 | -22.75 | ＜0.001 |
| Pain(F) | 1.03 | 1.00 |  | 2.21 | 0.90 |  | -1.14 | 1.25 | 0.92 | -26.39 | ＜0.001 |
| Pain(S) | 0.89 | 1.06 |  | 2.20 | 0.97 |  | -1.18 | 1.11 | 1.06 | -27.01 | ＜0.001 |
| Pain(I) | 1.24 | 1.04 |  | 1.59 | 1.11 |  | -1.30 | 1.20 | 1.09 | -9.10 | ＜0.001 |
| Insomnia(S) | 1.20 | 1.11 |  | 1.51 | 1.20 |  | -0.34 | 0.93 | 0.37 | -7.26 | ＜0.001 |
| Insomnia(I) | 1.65 | 0.97 |  | 2.68 | 0.80 |  | -0.31 | 1.06 | 0.29 | -30.94 | ＜0.001 |
| Fatigue(S) | 1.64 | 1.05 |  | 2.73 | 0.86 |  | -1.03 | 0.83 | 1.24 | -30.01 | ＜0.001 |
| Fatigue(I) | 1.25 | 0.92 |  | 1.34 | 0.81 |  | -1.09 | 0.90 | 1.21 | -2.57 | 0.01 |
| Anxious(F) | 1.24 | 0.95 |  | 1.38 | 0.82 |  | -0.09 | 0.84 | 0.10 | -3.76 | ＜0.001 |
| Anxious(S) | 1.15 | 1.03 |  | 1.16 | 0.91 |  | -0.14 | 0.90 | 0.15 | -0.26 | 0.793 |
| Anxious(I) | 1.50 | 0.97 |  | 1.53 | 0.88 |  | -0.01 | 0.92 | 0.01 | -0.69 | 0.49 |
| Sad(F) | 1.29 | 0.94 |  | 1.32 | 0.79 |  | -0.02 | 0.81 | 0.03 | -0.93 | 0.355 |
| Sad(S) | 1.10 | 1.01 |  | 1.15 | 0.92 |  | -0.03 | 0.87 | 0.04 | -1.17 | 0.243 |
| Sad(I) | 1.65 | 1.17 |  | 2.15 | 1.06 |  | -0.04 | 0.93 | 0.05 | -11.02 | ＜0.001 |
|  | | | | | | | | | | | |

Abbreviations: F, Frequency; I, Interference with daily activities; PRO-CTCAE, patient-reported outcomes version of the common terminology criteria for adverse events; S, Severity; SD, standard deviation; SRM, Standardized response means
